# Inhalable Antimicrobials for Treatment of Bacterial Biofilm-Associated Sinusitis in Cystic Fibrosis Patients: Challenges and Drug Delivery Approaches

**DOI:** 10.3390/ijms17101688

**Published:** 2016-10-09

**Authors:** Sylvia Natalie Kłodzińska, Petra Alexandra Priemel, Thomas Rades, Hanne Mørck Nielsen

**Affiliations:** Department of Pharmacy, Faculty of Health and Medical Sciences, University of Copenhagen, Universitetsparken 2, DK-2100 Copenhagen, Denmark; sylvia.klodzinska@sund.ku.dk (S.N.K.); psj748@ku.dk (P.A.P.); thomas.rades@sund.ku.dk (T.R.)

**Keywords:** biofilm, drug delivery, formulation design, devices, inhalable drugs, sinusitis, cystic fibrosis

## Abstract

Bacterial biofilm-associated chronic sinusitis in cystic fibrosis (CF) patients caused by *Pseudomonas aeruginosa* infections and the lack of available treatments for such infections constitute a critical aspect of CF disease management. Currently, inhalation therapies to combat *P. aeruginosa* infections in CF patients are focused mainly on the delivery of antimicrobials to the lower respiratory tract, disregarding the sinuses. However, the sinuses constitute a reservoir for *P. aeruginosa* growth, leading to re-infection of the lungs, even after clearing an initial lung infection. Eradication of *P. aeruginosa* from the respiratory tract after a first infection has been shown to delay chronic pulmonary infection with the bacteria for up to two years. The challenges with providing a suitable treatment for bacterial sinusitis include: (i) identifying a suitable antimicrobial compound; (ii) selecting a suitable device to deliver the drug to the sinuses and nasal cavities; and (iii) applying a formulation design, which will mediate delivery of a high dose of the antimicrobial directly to the site of infection. This review highlights currently available inhalable antimicrobial formulations for treatment and management of biofilm infections caused by *P. aeruginosa* and discusses critical issues related to novel antimicrobial drug formulation design approaches.

## 1. Introduction

**Figure 1:**
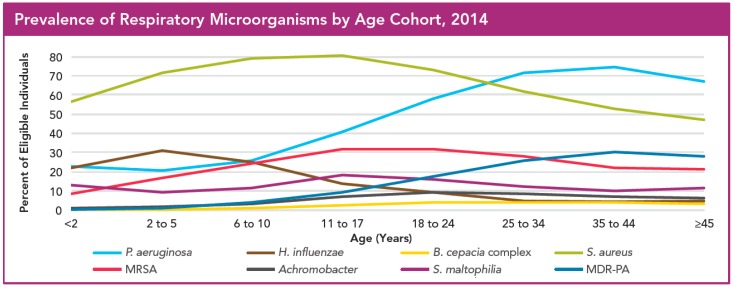
Prevalence of respiratory pathogens in cystic fibrosis patients by age cohort in 2014. Data are retrieved from the US CF Patient Registry and represent a cross-sectional analysis of the respiratory culture samples from patients in 2014. MDR-PA: Multi-drug resistant *P. aeruginosa*; MRSA: Multi-drug resistant *S. aureus*. Reproduced with permission from 2014 Patient Registry Annual Data Report [18].

Cystic fibrosis (CF) is a genetic disorder in Caucasians caused by mutations in the CF transmembrane conductance regulator (*CFTR*) gene [1]. *CFTR* is expressed in epithelia throughout the body, and affects secretory functions in many organs, such as the lungs, gut and sweat glands [2]. The *CFTR* gene encodes for the expression of a membrane-bound chloride channel in the lung epithelium; thus, a mutation in the *CFTR* gene results in decreased chloride and water secretion [3]. The altered fluid electrolyte composition of respiratory airway secretions results in abnormally viscous lung fluids leading to impaired mucociliary clearance of the fluid from the lungs [2,4]. This change in lung fluid characteristics provides better microbial growth conditions thereby facilitating more frequent and recurrent bacterial infections and neutrophil-rich inflammations [3]. The thickening of lung fluids and concurrent infections may occur already in pediatric CF patients and are often the cause of respiratory failure and death in CF patients at a young age [2]. The most frequently isolated bacterial pathogens from CF patient lungs are *Haemophilus influenza*, *Staphylococcus aureus* and *Pseudomonas aeruginosa* [3,5]. Lung infections with *P. aeruginosa* increase the mortality risk in this patient group almost three times as compared to CF patients without a *P. aeruginosa* infection [6]. The number of *P. aeruginosa* infections increases drastically to over 70% in patients aged 25–34 years (Figure 1) and chronic lung infections with this pathogen cause a more rapid pulmonary function decline, contributing to early death of CF patients [3,7]. It is estimated that 80% of CF patient mortality is directly connected to lung diseases, which are mainly the result of chronic airway infections [4]. *P. aeruginosa* is thus considered the most relevant bacterial pathogen to combat in CF patients. Infections with the bacterium at an early disease stage are usually temporary, as the infections may be cleared either spontaneously or after a course of treatment with anti-pseudomonas antimicrobials [8]. These infections are generally caused by *P. aeruginosa* pathogens that are susceptible to a wide-range of antimicrobials, and these transient infections are not related to long-term deterioration of pulmonary function [4,9]. However, one of the most prominent features of *P. aeruginosa* is the ability to develop resistance to essentially all antimicrobials through a range of genetic mutations in combination with production of a biofilm matrix around bacterial colonies [3]. Aiming at eradicating this bacterium and limiting the risk of resistance development, many European CF centers and the American Cystic Fibrosis Foundation (CFF) recommend aggressive antimicrobial treatment with inhaled tobramycin as soon as *P. aeruginosa* is first detected [3,4,8,10,11]. Treatment soon after diagnosis has shown to be successful in delaying occurrence of chronic infections with *P. aeruginosa* and thus improving quality of life for CF patients [3,12,13,14]. Other studies have shown that the sinuses in CF patients constitute a reservoir for *P. aeruginosa* from which the bacteria can be transmitted into the lungs, even after the initial infection in the lungs has been cleared [6,15,16,17]. Although the importance of eradication of *P. aeruginosa* from sinuses has been recognized, long-term clinical trials for treatments of CF sinus infections are still lacking.

This review highlights available and emerging antimicrobial drug delivery strategies for treatment of *P. aeruginosa* infections in the lungs of CF patients by using inhalable (Inhalation is defined by the drawing in of a substance into the lungs. In this review, the definition of inhalable antimicrobials includes nebulized treatments) therapies. A concise description of the nature of CF sinus infections and the bacteria that cause them along with an overview of the challenges to efficiently deliver drugs directly to the infection site in the sinuses are given. This is supplemented by an overview of current and emerging trends towards optimal drug formulation design for inhaled antimicrobials to treat *P. aeruginosa* infections featuring advantages and challenges of the most promising drug delivery systems.

## 2. *Pseudomonas* Infections in Cystic Fibrosis Patients

*P. aeruginosa*, which can be found in many man-made and natural water reservoirs [3,19], has been isolated from sputum samples in approximately 27% of pediatric CF patients [3], in whom it causes persistent and life-threatening infections. Sputum samples for bacterial culture analysis are collected from CF patients during regular control consultations usually every 3–6 months. The time point for the first detection of a culture positive for *P. aeruginosa* is denoted as “first infection”, though “first positive culture” would be more precise. The first positive culture from sputum usually occurs early in the patient’s life and although some patients may be able to clear the initial infection spontaneously, subsequent culture samples will be more frequently positive for *P. aeruginosa*. Once more than 50% of airway culture samples over the course of a year are positive for *P. aeruginosa*, the infection is considered chronic. Chronic infections are per definition difficult to eradicate, thus recent approaches aim at eradicating the initial infection in order to delay emergence of chronic infections [20].

Bacterial infections in the sinuses caused by *P. aeruginosa* are especially challenging for CF patients, as a *Pseudomonas* sinus infection is thought to spread easily from the sinuses into the lower respiratory tract and colonize the lungs [6,21]. The unified airway model considers the whole respiratory system as one functional unity, which consists of the nose, paranasal sinuses, pharynx, larynx, trachea, bronchi and distal lungs [15]. In most parts of the respiratory airway, the epithelium from the nose to the bronchi is composed of pseudostratified, ciliated, columnar epithelium [15]. This similarity in epithelial morphology is thought to be the reason for spreading of infections between upper and lower parts of the respiratory tract [5,15]. The unified airway model supports the idea that the sinuses may constitute a reservoir for the pathogen from where it spreads into the lower airways, where it can sustain lung infections [6,15,17]. This theory has also been explored in CF patients, from whom bacteria type, strain and antimicrobial susceptibility from both sinus and lung cultures were compared. This study showed that the type and strain of the pathogen from sinus and lung sputum were the same in the individual CF patient in 47% of all cases [22]. In another study, 73% of bacteria isolated from sinus cultures were also present in the lower respiratory tract cultures [23]. In a recent study, a majority of CF patients displayed sinus and lung infections with an identical genotype of *P. aeruginosa* [24]. All these reports suggest that the sinuses may act as a reservoir for bacterial infections in the lungs, and thus control of sinus infections should be a priority for improving lung health in CF patients. Indeed, studies indicate that aggressive treatment of sinus infections may positively affect the occurrence of lung disease and the quality of life for CF patients [8,10], but lack of clinical trials with long-term follow-up regarding medical and surgical interventions for sinus infections in CF patients make generalizations difficult [15].

## 3. Current Management and Treatment of Bacterial Infections in Cystic Fibrosis Patients

CF is a complex and multi-faceted genetic disease with types and severity of symptoms varying from patient to patient. Many factors, such as age of diagnosis, genotype and pancreatic insufficiency can influence a patient’s wellbeing and the course of the disease. Thus, patients may be stable for many years and show sudden periods of rapid progression of the disease. Care plans need to be carefully adjusted to individual needs of the patient. The CFF has prepared Clinical Practice Guidelines [11,25,26], which are followed by CF centers in the US. These guidelines highlight the importance of routine controls of patients, patient and family education and early intervention to slow disease progression. Three distinct phases of the disease regarding lung health are distinguished in CF patients: (i) early lung disease and initial infection with *P. aeruginosa*; (ii) chronic infection with the pathogen; and (iii) periodic exacerbations in pulmonary symptoms [27].

### 3.1. Antimicrobial Therapy to Treat Infections with Pseudomonas

The choice of antimicrobials should be based on two rules outlined by the CFF. First, antimicrobial agent selection should be supported by periodic isolation, identification of pathogens from lung sputum and review of the antimicrobial’s susceptibility profile for these pathogens; Second, use of antimicrobial agents without considerations of the rationale, clinical endpoint, and duration should be avoided [27].

Currently, inhaled antimicrobials are a standard recommendation for treatment of chronic lung infections with *P. aeruginosa* in CF patients [28]. The Food and Drug Administration (FDA) has approved inhalable drug formulations with tobramycin, aztreonam and azithromycin for this indication, while the European Medicines Agency (EMA) has additionally approved colistimethate sodium. Other inhalable antimicrobial drug formulations applied for this indication are being developed [3,28]. Antimicrobials are prescribed in all three phases of CF; the most aggressive treatment occurring at the time of first infection, with the aim of delaying the occurrence of chronic infection with *P. aeruginosa*. For this stage of the disease, the CFF recommends inhaled tobramycin [11]. During the second maintenance stage, inhalable formulations with tobramycin, aztreonam, azithromycin, or colistimethate sodium may be used, but due to insufficient amounts of data on the effects of inhaled colistimethate sodium in CF patients, use of the first three mentioned antimicrobials is preferred [26]. During periodic exacerbations, a combination of intravenous β-lactam and aminoglycoside antimicrobials are frequently administered, usually during hospitalization [25,27]. Other antimicrobials for this indication are currently being developed. As an illustration hereof, several clinical trials show encouraging results of inhaled fluoroquinolones for treating chronic infections in CF patients as well as for treating patients with bronchiestasis [28]. Further, six ongoing phase 3 trials are assessing the efficacy of inhaled levofloxacin and ciprofloxacin. Fosfomycin and aminoglycoside combinations are also being assessed in two ongoing phase 2 studies. This combination was shown to be effective for treating lung infections in CF patients and is now being investigated for treatment of ventilator-associated pneumonia [28].

Despite the fact that there are strong rationales to support the use of antimicrobials such as tobramycin, colistimethate sodium and aztreonam for treating *P. aeruginosa* lung infections in CF patients, large clinical trials for studying therapy of CF sinus infections are lacking [29]. However, a small double-blinded, placebo-controlled trial exploring the effect of nebulization of tobramycin to the sinuses was conducted [30]. Results from that study showed positive effects on the treatment of sinonasal symptoms as well as reduced presence of *P. aeruginosa* in nasal lavages from treated CF patients compared to patients treated with saline irrigations. Another small study applied nebulized antimicrobials for treating acute infections in chronic sinusitis in non-CF patients. Out of 11 *Pseudomonas* mono-infections, one was treated with tobramycin, five with levofloxacin, four with ceftazidime and one with ciprofloxacin. In total, 7 out of the 11 infections were considered to be cleared at the last control after three weeks of treatment and a three-month follow-up [31]. Application of antimicrobials post endoscopic sinus surgery has also been associated with a decreased recurrence of sinus exacerbations in CF patients and improved sinus health for two years following surgery [32,33].

### 3.2. Non-Antimicrobial Therapy to Treat Infections with Pseudomonas

The treatment of CF patients is mostly symptomatic, focusing on slowing disease progression and deferment of inevitable lung damage. A variety of drugs, such as mucolytics, bronchodilators and anti-inflammatory drugs are used for this purpose, often multiple times per day [2,5]. To the authors’ knowledge, none of these treatment regimens have been evaluated in randomized controlled trials [5]. Ibuprofen is used in anti-inflammatory therapy, which is essential in treating CF lung infections, to prevent or delay a decline in pulmonary function, tissue remodeling and tissue destruction, such as chronic airway inflammation, that is uniformly observed in CF patients [3]. Bronchodilators and/or mucolytic agents, such as recombinant human DNase (dornase alfa), and/or physiotherapy precede the administration of antimicrobials by nebulization, to partly alleviate the significant negative effect that infected CF sputum has on the activity of aerosolized medication. Nebulized saline is used for lung therapy in CF patients [34], whereas saline irrigations are more commonly used for sinusitis. Hypertonic nasal saline irrigations may improve mucociliary clearance and thereby help to remove thickened secretions and crusting, which accumulate due to the underlying CF pathogenesis [3,15]. Although trials on the application of saline irrigation in CF patients in comparison to antimicrobial treatment are lacking, the treatment is used and recommended based on the benefits demonstrated in non-CF patients treated with hypertonic saline irrigation to remove thickened secretions and crusting [35]. Both the squeeze bottle and neti pot are devices, which provide very good delivery to the paranasal sinuses [15]. Commonly, dornase alfa, hypertonic saline, and antimicrobials are used in combination to achieve the best treatment results, with 33% of all patients taking all three inhaled medications [18].

### 3.3. Endoscopic Sinus Surgery and Preventive/Post-Operative Use of Antimicrobials

**Figure 2:**
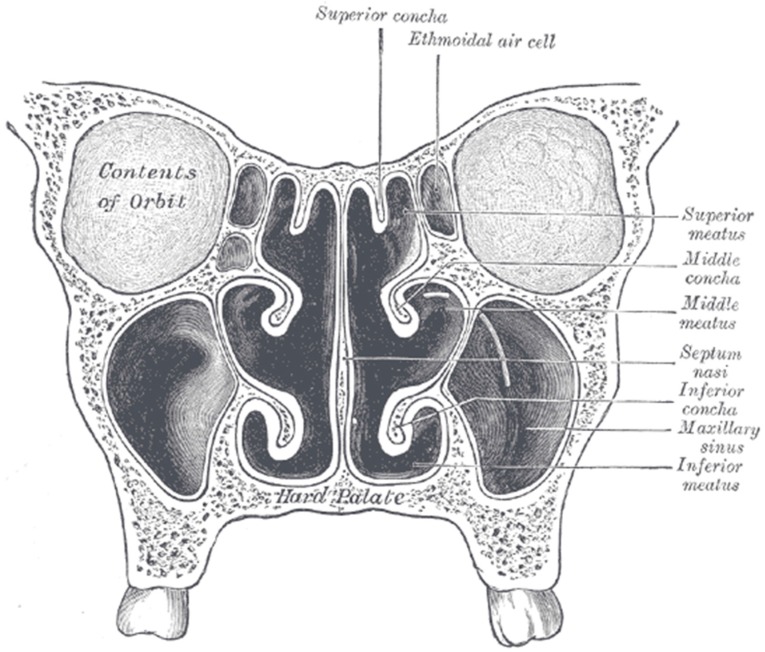
Coronal section of nasal cavities. Source Lewis (1918) Gray’s Anatomy 20th edition [37].

Endoscopic sinus surgery (ESS) is a surgery by which the middle meatus and maxillary ostium (between the middle meatus and the maxillary sinus) (Figure 2) are opened as wide as possible to facilitate later irrigations. It is carried out in patients with recurrent chronic sinusitis, when other treatments show not to be effective. ESS is also recommended for CF patients undergoing lung transplantation, in order to clean sinuses before lung surgery [32]. Treatment with antimicrobials is recommended post-ESS to prevent infection inthe sinuses, and warm saline irrigations twice daily with liquid containing tobramycin can be used. At present there are no other specific criteria for ESS in medical management of chronic sinusitis in CF patients. The decision to carry out this treatment is made by clinicians based on previous experiences and the specific patient complaints. Although a majority of CF patients show signs of sinusitis upon radiographic and endoscopic examination, only about 10% of patients report symptoms to their clinician [15]. This discrepancy between reported symptoms and observed symptoms makes it difficult to decide on relevant indications for surgical intervention. Thus, the most common indication for ESS is unresolved symptoms after a course of treatments with antimicrobials. However, more aggressive strategies to treat CF sinusitis patients without symptoms are considered increasingly favorable by clinicians in some centers as evidence points towards surgical management as a good solution for eradicating the bacterial reservoir in the sinuses and thus improving pulmonary therapy outcomes [32,36].

## 4. Antimicrobials for Inhalation: State-of-the-Art

The first reported attempt at aerosol delivery of antimicrobials was in 1940; however, the treatment resulted in poor tolerability by the patients and failed due to poor formulation properties since hyperosmolarity and preservatives in the aerosol caused bronchial irritation and bronchospasm in the CF patients. Another problem was the lack of suitable nebulizers, which would allow maximal delivery of the drug to the respiratory airway [38]. Twenty years later, Elek and Fleming developed a method for eliminating nasal carriage of bacteria in hospitalized infants; this was done by spraying a solution of methicillin into the air in the hospital nursery [39]. The researchers noticed a decrease in the frequency of *S. aureus* colonization among the infants, but instead the infants showed a tendency to become colonized with methicillin-resistant strains of coagulase-negative staphylococci [40]. A major breakthrough occurred in the 1990s when inhaled tobramycin was first evaluated in a number of studies, and subsequently in 1997 was approved by the FDA for treatment of *P. aeruginosa* infections in the lower airways of CF patients [38]. Since then, the interest in inhaled antimicrobial formulations for treatment of local infections has increased, especially for CF patients [2]. Currently, inhaled drugs are recommended for treatment and management of early and chronic *P. aeruginosa* infections as well as improvement of airway hydration, mucociliary action and mucus clearance by using mucolytic agents [2].

The general advantages of utilizing topical, compared to systemic, drug administration are the ability to achieve high drug levels at the target site in CF patient respiratory airways [41,42], limited systemic toxicity [41,42,43,44], fast onset of action, direct drug action on site, suitability for self-medication and the reduction of bacterial resistance as a consequence of bacteria eradication [41]. This is especially important when antimicrobials such as aminoglycosides are used as they show low penetration into the lungs when administered intravenously [45,46,47]. The pulmonary delivery route also prevents the exposure of the gastrointestinal microflora to the antimicrobial, avoiding side effects in the digestive system and decreasing the risk of bacterial resistance development [28]. Associated with the topical delivery route are also some potential disadvantages, such as uncertainty of the drug dose at infection site, local side effects such as coughing and respiratory airway narrowing, as well as possible systemic absorption across the lung tissue and drug interactions occurring in the lung [2,3]. Importantly, successful pulmonary drug delivery depends on the patient’s inhalation technique, the delivery device, and the drug delivery system.

The success of topical treatment in the respiratory tract is strongly dependent on the site and the extent of drug deposition, the penetration of the drug into the lung fluid, drug interaction with the cell target and its ability to escape macrophage clearance [2]. The ability of the drug delivery system or the drug to penetrate the lung fluid, mucus as well as bacterial biofilms is especially important for efficient delivery of antimicrobials to their target, the bacteria. The negatively-charged alginate matrix produced by the bacteria is known to slow down drug penetration and possibly entrap (especially positively charged) antimicrobials in the biofilm, thereby decreasing drug availability in the proximity of bacteria. The physico-chemical properties of the antimicrobials, such as charge and degree of lipophilicity, may be shielded when encapsulated into particulate systems. Neutral charge of the particles and an aerodynamic diameter <5 µm are especially important; the former to decrease the adherence of the drug delivery system to the bacterial biofilm, while the latter ensures that the particles will deposit in the lower respiratory tract upon aerosolization [43]. Understanding the interactions between the inhaled drug as well as drug delivery system and the CF lung environment is thus crucial for designing new drug formulations with a therapeutic effect in CF patients [2].

Antimicrobial drug formulations for pulmonary delivery can be categorized in two main groups: liquid formulations for aerosolization, which require applying nebulizers for administration, and dry powders, which are delivered via dry powder inhalers (DPIs). Patients generally prefer DPIs [43], however, due to decline of lung function and inspiratory volume force, nebulization may be necessary at later disease stages.

### 4.1. Liquid Formulations

Solutions and suspensions for delivery as aerosols via nebulizers are the most common formulations applied for treatment of CF-associated lung infections. Some lyophilized powders containing antimicrobials that are unstable in liquid formulations, such as aztreonam and colistimethate sodium, have been developed and are intended for reconstitution with saline immediately prior to use [2].

Nebulized antimicrobials against *P. aeruginosa* infections have been developed as an alternative to intravenous administration, allowing the delivery of an antimicrobial compound directly to the site of infection, thus improving efficacy and reducing systemic toxicity [43]. Amongst others, the CFF encourages the long-term use of inhaled tobramycin and dornase alfa in patients with moderate to severe lung disease to improve pulmonary function and quality of life. Inhalable tobramycin, azithromycin, colistimethate sodium and aztreonam lysine formulations are other available therapies for treatment of *P. aeruginosa* infections in the lungs (Table 1) [43]. Use of the Tobramycin Inhalation Solution (TIS) has been studied in some detail. The recommendation for using TIS is a “1 month on, 1 month off” treatment regimen, as continuous therapy with TIS has been shown to increase development of bacterial resistance [20,43]. Maintenance therapy with TIS in this recommended cycle reduces exacerbations and improves pulmonary function. Most importantly, therapy with nebulized tobramycin to treat first infection with *P. aeruginosa* delayed the emergence of chronic infections in approximately two-thirds of CF patients included in some studies [12,13,14]. Nebulized aztreonam lysine has shown similar efficacy to TIS maintenance therapy, also effectively delaying the onset of chronic infections and the need for antipseudomonal antimicrobials for lung exacerbations in CF patients. Nebulized colistimethate sodium may be used for continuous *P. aeruginosa* growth suppression in maintenance therapy, although it is not preferred by the CFF due to lack of results from clinical trials.

Liquid formulations with antimicrobials are administered to CF patients mainly by using nebulizers, requiring inhalation for approximately 30 min at each dosing. Additionally, using nebulizers requires rigorous cleaning after each use, which may be multiple times a day, in order to decrease the risk of contamination. As nebulizers are bulky and not easy to carry, their use is rather inconvenient [43].

Although clinical studies of TIP and Colobreathe^®^ have shown non-inferiority of the DPI compared to the nebulizer, it is important to remember that there are many patient- and device-related differences between the two inhalation methods that may influence the efficacy of the treatment. It is doubtful that for all patients the efficacy of DPI-administered antimicrobials is the same as that of antimicrobials administered by nebulization [43].

### 4.2. Dry Powders

Recently, a tobramycin inhalation powder (TIP) was developed as an alternative to TIS. Tobramycin is a positively charged antimicrobial, and its efficacy is strongly impaired by drug binding to negatively charged alginate in bacterial biofilms in the CF patient respiratory tract [48,49]. Lower doses of the dry powder (112 mg) given twice daily have been reported to be comparable to dosing of 300 mg antimicrobial in a liquid formulation (TIS), both resulting in therapeutically active concentrations in infected CF patient lungs [2]. A dry powder formulation for colistimethate sodium (Colobreathe^®^) has also been developed as an alternative to the pulmonary aerosol formulation. Clinical trials with TIP and Colobreathe^®^ have shown to be non-inferior to the nebulized formulations [43]. Currently, other antimicrobials, such as ciprofloxacin or levofloxacin for eradicating *P. aeruginosa* and vancomycin or clarithromycin for clearing *S. aureus* are also in development as dry powders for inhalation using DPIs [43].

The use of DPIs has important advantages over using nebulizers for administration to patients: the administration time is much shorter, no rigorous cleaning or maintenance of the device is needed, the inhalers are small and the dry powder capsules are conveniently packaged in sealed blisters, which do not require refrigeration as some liquid formulations. DPIs are thus significantly more convenient for the patient than nebulizers, making the inhaled dry powders the formulation of preference for CF patients [43].

### 4.3. Emergence of Resistance to Inhaled Antimicrobials and Other Adverse Effects

The main concern with regards to long-term administration of inhaled antimicrobials is the risk of development of antimicrobial resistance. Primarily, resistance is thought to be caused by sub-inhibitory concentrations of antimicrobial in the deep lung, resulting from insufficient or inadequate dosing. However, administration via inhalation results in drug deposition in the deep airways at concentrations which are significantly higher than the concentrations in lung resulting from intravenous administration of the antimicrobial [50]. As a result, inhaled antimicrobials are more likely to provide concentrations above both the MIC and mutant prevention concentration (MPC) for intrapulmonary infections, whereas systemic administration of the same antimicrobial would result in lower concentrations in the lung fluid. This supports the theory that systemic administration of antimicrobials may increase the risk of sub-inhibitory concentrations in the deep lungs and as a result, increase the risk of antimicrobial resistance development [51]. The MPC is defined as the concentration that severely restricts the selection of resistant mutants present in high density populations during antimicrobial treatment [52] and would normally be much higher than the MIC, thus higher exposure concentrations are necessary to reach the MPC.

Using inhalable antimicrobials may thus support resistance prevention rather than inducing it. Despite this, data supporting increased occurrence of resistant bacteria as a result of long-term treatment with inhaled tobramycin in CF patients has been reported [53]. However, this did not seem to diminish the efficacy of the antimicrobial as the dose administered was high enough to overcome both the binding to eDNA and increased resistance of bacteria in the lung [3]. However, a widespread resistance to intravenously administered tobramycin would pose a significant clinical problem [54]. A Cochrane review from 2011 reports on the use of inhaled tobramycin in CF patients stating that although resistance to antimicrobials increased more in the group treated with inhalable tobramycin, as compared to the placebo group, the lung function was improved and exacerbations of lung infection were less in the antimicrobial-treated group [29]. Aerosolized tobramycin has shown to be safe without resulting in renal or audiologic complications after both long-term and short-term use [3,4,29]. In addition, colistimethate sodium is generally well tolerated by patients [3] and resistance to colistimethate sodium is rare [55] although bronchoconstriction following inhalation of colistimethate sodium is quite common in CF patients, especially those suffering from asthma [56]. Colistin sulfate, on the other hand, is not advisable for treatment of CF patients due to severe side effects [57].

## 5. Formulation Approaches for Pulmonary Delivery of Antimicrobials

The wide distribution of multi-drug resistant bacteria has attracted research on novel antimicrobial formulations for management and eradication of *P. aeruginosa* chronic infections [2].

Nanomedicines have been used extensively for the formulation of antimicrobials targeting *P. aeruginosa*, especially for prevention of biofilm formation and eradication of existing biofilms with this pathogen. Nanoparticles based on both lipids (such as dipalmitoyl phosphatidylcholine, phosphatidylcholine, cholesterol) and polymers (poly (lactic-*co*-glycolic) acid, alginate and chitosan) have been widely utilized for this purpose (Table 2) as they can be administered either suspended in liquid form or as a dry powder suitable for inhalation. The advantages of these particulate systems include their compatibility with the human body, the wide variety of materials and surface modifications possible to implement, the possibility for controlled drug release, the potential for incorporating both lipophilic and hydrophilic drug compounds and the possibility of reducing potential toxicity and side effects of the antimicrobial [58]. Both lipid- and polymer-based particles have been shown to be effective in improving antimicrobial delivery into biofilms by shielding the antimicrobial from binding to the biofilm matrix, by protecting against enzymatic degradation and by increasing the contact time between the bacteria and the antimicrobial. Lipid particles have also been shown to fuse with the bacterial membrane, allowing direct delivery of the antimicrobial to the bacteria [58]. CF sputum contains not only mucins, but also large quantities of DNA and filamentous actin released by necrotic host cells and bacteria during infection and inflammation [59]. Due to the presence of negative charges on the mucin molecules, the polymers can interact with and complex cationic molecules. Some studies have shown significant interactions between mucin and charged nanoparticles [59,60]. For successful delivery of antimicrobials to the respiratory epithelium, it is therefore critical to overcome the physical and chemical barriers of CF sputum. Some lipid- and polymer-based particulate formulations, aimed for delivery of antimicrobials to *P. aeruginosa*, are listed in Table 2.

## 6. Delivery to Sinuses

Both inflammation and impaired mucociliary clearance contribute to blockage of sinus drainage creating a favorable environment for bacterial infection in the sinuses [42]. Although eradication of *P. aeruginosa* from sinonasal reservoirs is considered to delay the occurrence of pulmonary chronic infections, no formulations specifically aiming at administration to the sinuses have yet been developed. For sinus delivery, three important administration sites are relevant as targets for aerosolized antimicrobials (Figure 2): (i) the middle meatus, which is the main site of sinus drainage; (ii) the superior and posterior regions of the nasal cavity; and (iii) the maxillary and ethmoid sinuses [61].

As previously outlined with regards to delivery of antimicrobials to the lungs, also the delivery of antimicrobials for treatment of *P. aeruginosa*-associated sinusitis has advantages compared to systemic therapy; it generally minimizes the risk of systemic side effects, including the risk of development of antimicrobial resistance by avoiding deposition of antimicrobials at non-target areas and allows for a high local drug concentration at the site of action. However, further insight into the properties needed for the drug delivery system to reach these anatomical cavities upon nebulization or dry powder dosing utilizing a certain device is lacking. The paranasal sinuses are virtually non-ventilated, poorly perfused hollow cavities protected from entry of foreign matter by efficient particle filtration in the nose [62]. It is thus very difficult to achieve effective sinonasal targeting of drugs nebulized into the nose [42]. The maxillary sinuses are connected with the nasal fossa via the maxillary ostia, which are about 1–5 mm in diameter and 10–15 mm in length. Some in vivo and in vitro studies have shown that aerosolized particles can reach and accumulate in the paranasal sinuses, but only at low concentrations [42]. Those studies emphasize the three most important aspects affecting aerosol deposition in the sinuses, which are diameter and length of the maxillary ostium, the pressure of the aerosol and the airborne particle size.

Although nasal drug delivery for sinusitis treatment is extensively recommended by clinicians, optimizing formulations to reach the sinuses is necessary to take full advantage of this method of delivery [42]. 

## 7. Devices for Drug Delivery to the Sinuses

Overall, there seems to be significant evidence supporting the use of antimicrobials administered to the sinuses for treatment of sinusitis [95], and, currently, nebulizers are the only devices present on the market for delivery of antimicrobials to the sinuses [96]. In a study from 2008, various devices for delivery of antimicrobials to the sinuses were compared, and no evidence was found to recommend the use of nasal sprays, whereas some evidence was found that nasal irrigations and delivery by nebulization were effective. In the latter case, it was found that an important parameter was that the particle size should be <5 µm [62,95]. The effectiveness of the drug administered by nasal sprays depends on the mucociliary clearance, which is crucial for drug distribution and subsequent elimination. However, mucociliary clearance may be impaired in CF patients with sinusitis. In addition, nasal sprays only deliver a low fraction of the drug beyond the nasal valve due to the device designs [97], and most of the deposited dose is quickly eliminated via the digestive tract [98]. On the other hand, nasal nebulization improves drug deposition below the nasal valve compared to the deposited amount achieved by using nasal sprays [97]. Nasal sonic jet nebulizers applying a frequency of 100 Hz have been developed to improve aerosol deposition in the sinuses compared to regular nasal jet nebulizers [99]. The 100 Hz sound generated by the sonic jet nebulizer creates a positive pressure from the ostium to the sinus allowing gas exchange with the sinus similar to a Helmholtz resonator [42,100]. Both in vitro and in vivo experiments have demonstrated the benefit of applying sound for improved sinus ventilation and aerosol deposition [100,101,102,103], and it has been shown that it may increase aerosol deposition into the sinuses by a factor two [42,104]. Nasal sonic jet nebulizers with sound effect are therefore considered the best option for targeting aerosols to the sinus infection site [96]. However, a disadvantage of using nasal jet nebulizers for targeting the sinuses is that they also deliver a relatively large volume, from 33% to 58%, of the aerosol into the lungs [97], which in itself increases the risk of side effects, influences the efficiency of the treatment of infections in the sinuses and results in a variable dose–response relationship [96,105]. Similar levels of drug deposition in the lungs were obtained from utilizing a jet nebulizer without sound effect and a nasal sonic jet nebulizer with sound effect, indicating that the sound effect did not have a significant influence on the level of aerosol deposition in the lungs [96]. In contrast, a new type of mesh nebulizer intended for nasal administration of drugs considerably reduced the inhaled fraction of the aerosol deposited in the lungs. While the same particle size is produced by the sonic jet and mesh nebulizer, they do not create the same aerosol flow path through the sinonasal cavity. The nasal sonic jet nebulizer delivers the drug by inhalation through the nostrils of the patient. The nebulized particles reach the lungs via the nasal cavity during the patient’s breathing. In contrast, the special compressor in the mesh nebulizer administers a constant air flow via one nostril and aspirates the same air flow via the other nostril. As a result the aerosol is administered though one nostril and aspirated through the other, causing the soft palatine to close the nasal cavity, which prevents aerosol penetration into the lungs. As a consequence, during inhalation with the mesh nebulizer, the patient has to breathe through the mouth [62,96].

## 8. Concluding Remarks

Recognizing the influence of infections in the sinuses in seeding bacteria to the lung of CF patients has sparked interest in improving treatment of sinus infections in these patients. Comprehensive individualized therapies including the use of mucolytics, anti-inflammatory agents, antimicrobials—and to the extent needed—endoscopic sinus surgery are currently recommended. Randomized, controlled clinical trials with long-term follow-up are needed to investigate efficacy of medical and surgical interventions for sinus disease in CF patients. In this review, a variety of inhalable formulations with antimicrobials not directly targeted to eradicate infections in the sinuses have been described. Some of these (Table 1) have been successfully nebulized for delivery of drug to the lungs and are now marketed for this indication. These formulations might thus be considered as applicable for treating sinus infections. The morphological similarities of the tissue in the upper respiratory tract and the sinonasal tissue, in terms of cell lining and sputum composition, support the use of the antimicrobial drugs currently applied by inhalation to the lungs also for targeting the bacteria and biofilm in the sinuses. Although there is still a shortage of studies evaluating inhaled antimicrobials targeting the respiratory airways and in particular the sinuses, the importance of sinuses as a target is increasingly recognized, and the interest in using inhalable formulations against infections in both CF patients and non-CF patients is increasing.

## Figures and Tables

**Table 1:** Inhaled antimicrobials marketed or undergoing clinical trials for treatment of *P. aeruginosa* infections in CF patients. Adapted from [2,43].

Drug	Product Name	Development Status	Formulation
Tobramycin	Tobi^®^, Bramitob^®^	Marketed	LF, DP
Levofloxacin	Aeroquin^®^	Phase III (NCT01270347, NCT01180634) *	LF
Aztreonam lysine	Cayston^®^	Marketed	LF
Colistimethate sodium	Promixin^®^, Colobreathe^®^	Marketed	LF, DP
Azithromycin	Zithromax^®^	Marketed	
Amikacin	Arikayce™	Phase III (NCT01315678) *	LF
Ciprofloxacin	Cipro Inhale^®^	Phase II (NCT00645788) *	DP

LF: Liquid formulation, DP: Dry powder. * More information on clinicaltrials.gov.

**Table 2:** Examples of lipid and polymeric antimicrobial formulations under development for *P. aeruginosa* infections.

Antimicrobial	Composition	Form	In Vitro/In Vivo Model	Improved Antimicrobial Effect?	Mechanism of Action	References
Tobramycin	DSPC/DMPG (10:1 molar ratio)	LF	Intratracheal administration in healthy rats or rats chronically infected with *P. aeruginosa* in agar beads	Yes	Increased residence time of tobramycin in lungs	[63]
Tobramycin	DPPC/DMPG (10:1 to 15:1 molar ratio)	LF	Intratracheal administration in rats chronically infected with *P. aeruginosa* in agar beads	Yes	Increased residence time of tobramycin in lungs	[64]
Tobramycin bismuth-thiol	DSPC/cholesterol (2:1 molar ratio)	LF	Clinical strains of *P. aeruginosa* from CF sputum	Yes	Disturbs bacterial membrane integrity and protects against binding to eDNA	[65,66,67]
Tobramycin	Cholesterol/lecithin	DP	Pilot study in CF patients receiving dose via a breath-actuated DPI	N/A	Improved lung deposition	[68,69]
Tobramycin	Alginate/chitosan, DNase	LF	CF patient sputum *Galleria mellonella* PA01 infection model	Yes, complete eradication	Prolonged residence time at infection site, improved penetration through CF sputum	[70]
Tobramycin	Precirol^®^ ATO 5 50:50 Compritol^®^ 888 ATO: Precirol^®^ ATO 5/Miglyol1812	LF	Clinical strains of *P. aeruginosa* from CF sputum Artificial CF sputum model Cytotoxicity in H441 and A549 cells biodistribution study in mice	Yes	Sustained release of antimicrobial, fusion with bacterial membrane	[71]
Tobramycin	DPPC/DMPG	DP	Clinical strains of *P. aeruginosa*	Yes	Fusion with bacterial membrane	[72,73,74]
Tobramycin	DPPC/DMPG DSPC/DMPC	LF	Intratracheal administration rats chronically infected with *P. aeruginosa* in agar beads	Yes, complete eradication	Fusion with bacterial membrane (in vivo study)	[75]
Tobramycin Polymyxin B	DMPC/cholesterol DPPC/cholesterol	LF	Clinical strains of *P. aeruginosa* in CF sputum	Yes Yes	Protection against binding to eDNA and degradation	[76]
Tobramycin Amikacin Gentamicin	DPPC/cholesterol	LF	Laboratory strains of *P. aeruginosa*	Yes, complete eradication	Fusion with bacterial membrane	[77]
Tobramycin Gentamicin Amikacin	DMPC/cholesterol	LF	Clinical strains of *P. aeruginosa* from CF sputum	Not in the presence of mucin and CF sputum	Binding of the liposomes to mucin, alginate or sputum components	[78]
Gentamicin	PLGA	LF	Laboratory strains of *P. aeruginosa* 96-h peritoneal murine infection model	Yes	Controlled/ sustained release of antimicrobial	[79]
Gentamicin	DPPC/DMPG (ratio 10:1)	LF	Clinical strains of *P. aeruginosa* from CF sputum Cytotoxicity in A549 cells	Yes	Quorum sensing reduction, reduced binding to eDNA	[80]
Gentamicin	DMPC/cholesterol DPPC/cholesterol DSPC/cholesterol	LF	Clinical strains of *P. aeruginosa*	Yes	Protection of antimicrobial against degradation or fusion with bacterial membrane	[81]
Gentamicin Ciprofloxacin	PC/cholesterol/DOTAP PC/DOPE/DOTAP	LF	Clinical and laboratory strains of *P. aeruginosa*	No	Reduced binding to non-target materials	[82]
Clarithromycin	DDAB/DPPC/cholesterol DCP/DPPC/cholesterol DPPC/cholesterol	LF	Clinical strains of *P. aeruginosa*	Yes, complete eradication	Electrostatic attraction possibly followed by fusion, protection of the antimicrobial	[83]
Ciprofloxacin	HSPC/cholesterol	DP	Phase II clinical trials (Lipoquin^®^) for non-CF infections with *P. aeruginosa*	Yes	Sustained release at site of infection	[84]
Ciprofloxacin	PLGA, poly(lysine), DNase	LF	*P. aeruginosa* planktonic and biofilms Cytotoxicity in J774 cells	Yes	Biofilm formation prevention, improved penetration through biofilm	[85]
Ciprofloxacin	PEG/gelatin	LF	Clinical strains of *P. aeruginosa*	Yes	Sustained release of antimicrobial	[86]
Levofloxacin	Chitosan	DP	Clinical strains of *P. aeruginosa*	No	Immediate release at site	[87]
Levofloxacin	PLGA PLGA/PC	LF	*P. aeruginosa* planktonic and in biofilm	Yes, but to a lower extent	PLGA/PC particles could enhance antimicrobial	[88]
Colistimethate sodium	Precirol^®^ ATO 5	LF	Clinical strains of *P. aeruginosa* from CF sputum Cytotoxicity in H441 and A549 cells	Yes	Sustained release of antimicrobial	[89]
Colistimethate sodium	Precirol^®^ ATO 5/Miglyol^®^ 812	LF	Clinical strains of *P. aeruginosa* from CF sputum Cytotoxicity in H441 and A549, biodistribution study in mice	Yes	Sustained release of antimicrobial	[89]
Colistimethate sodium	Precirol^®^ ATO 5/Miglyol^®^ 812	LF	Clinical strains of *P. aeruginosa* from CF sputum	Yes, but only in biofilms	Reduced binding to non-target materials, improved delivery to proximity of bacteria in biofilm	[90]
Colistin	PLGA/PVA/chitosan	DP	Artificial mucus Laboratory strains of *P. aeruginosa*	Yes	Sustained release of antimicrobial	[91]
Netilmicin	PLGA/dextran sulfate	LF	Cytotoxicity in CFBE 41o- cells, Laboratory strains of *P. aeruginosa*	No	Protection against binding to eDNA	[92]
Azithromycin	DPPC/cholesterol (6:1 molar ratio)	LF	Clinical strains of *P. aeruginosa* Cytotoxicity on erythrocytes and A549 cells	Yes	Attenuated production of virulence factors and reduced bacterial mobility	[93]
Vancomycin	DPPC/cholesterol DPPC/DOPE/CHEMS	LF	Clinical strains of *P. aeruginosa*	Yes	Fusion with bacterial membrane	[94]

A549 = human lung carcinoma; CF = cystic fibrosis; CFBE41o- = human bronchial epithelial cells; CHEMS = cholesteryl hemisuccinate; DCP = dichlorophenol; DDAB = didecylmethylammonium bromide; DMPC = dimyristoyl-sn-glycero-3-phosphocholine; DMPG = dimyristoyl phosphatidyl-glycerol; DNase = deoxyribonuclease; DOPE = dioleoylphosphatidylethanolamine; DOTAP = dioleoyloxy-3-trimethylammonium-propane; DP = dry powder; DPPC = dipalmitoyl phosphatidylcholine; DSPC = distearoyl phosphatidylcholine; eDNA = extracellular deoxyribonucleic acid; H441 = human lung papillary adenocarcinoma cells; HSPC = hydrogenated soybean phosphatidylcholine; J774 = murine macrophage cells; LF = liquid formulation; N/A = not available; PC = phosphatidylcholine; PEG = poly (ethylene glycol); PLGA = poly (lactic-*co*-glycolic) acid; PVA = polyvinyl alcohol.

## Abbreviations

A549	Human lung carcinoma cell line
CF	Cystic fibrosis
CFBE41o-	Human bronchial epithelial cell line
CFF	Cystic Fibrosis Foundation
CFTR	Cystic fibrosis transmembrane conductance regulator
CHEMS	Cholesteryl hemisuccinate
DCP	Dichlorophenol
DDAB	Didecylmethylammonium bromide
DMPC	Dimyristoyl-sn-glycero-3-phosphocholine
DMPG	Dimyristoyl phosphatidyl-glycerol
DNase	Deoxyribonuclease
DOPE	Dioleoylphosphatidylethanolamine
DOTAP	Dioleoyloxy-3-trimethylammonium-propane
DP	Dry powder
DPI	Dry powder inhaler
DPPC	Dipalmitoyl phosphatidylcholine
DSPC	Distearoyl phosphatidylcholine
eDNA	Extracellular deoxyribonucleic acid
FDA	Food and Drug Administration
EMA	European Medicines Agency
ESS	Endoscopic sinus surgery
H441	Human lung papillary adenocarcinoma cell line
HSPC	Hydrogenated soybean phosphatidylcholine
J774	Murine macrophage cell line
LF	Liquid formulation
N/A	not available
PC	Phosphatidylcholine
PEG	Poly (ethylene glycol)
PLGA	Poly (lactic-*co*-glycolic) acid
PVA	Polyvinyl alcohol
TIS	Tobramycin inhalation solution
TIP	Tobramycin inhalation powder
